# Mannose-binding lectin suppresses macrophage proliferation through TGF-β1 signaling pathway in Nile tilapia

**DOI:** 10.3389/fimmu.2023.1159577

**Published:** 2023-05-16

**Authors:** Liangliang Mu, Xiaoxue Yin, Hao Bai, Jiadong Li, Li Qiu, Qingliang Zeng, Shengli Fu, Jianmin Ye

**Affiliations:** ^1^ Guangzhou Key Laboratory of Subtropical Biodiversity and Biomonitoring, Institute of Modern Aquaculture Science and Engineering, School of Life Sciences, South China Normal University, Guangzhou, Guangdong, China; ^2^ Guangdong Provincial Engineering Technology Research Center for Environmentally-Friendly Aquaculture, School of Life Sciences, South China Normal University, Guangzhou, Guangdong, China

**Keywords:** Nile tilapia, mannose-binding lectin, macrophage, cell proliferation, TGF-β1 signaling pathway

## Abstract

Mannose-binding lectin (MBL) is a multifunctional pattern recognition molecule, which not only mediates the recognition of pathogenic microorganisms and their products, playing an important role in innate immune defense, but also participates in adaptive immune responses of mammalian. However, it’s related immune mechanism remains limited, especially the regulation of cell proliferation in early vertebrates. In this study, OnMBL was found to bind to kidney macrophages (MФ) from Nile tilapia (*Oreochromis niloticus*). Interestingly, OnMBL was able to reduce the proliferation of activated-MФ by regulating the cell cycle, arresting a large number of cells in the G0/G1 phase, and increasing the probability of apoptosis. More importantly, we found that the inhibition of cell proliferation by OnMBL was closely related to the evolutionarily conserved canonical transforming growth factor-beta 1 (TGF-β1) signaling pathway. Mechanistically, OnMBL could significantly increase the expression of TGF-β1, activate and regulate the downstream Smad-dependent pathway to reduce the MФ proliferation, thereby maintaining cellular homeostasis in the body’s internal environment. This study represents the first description regarding the regulatory mechanisms of the MBL on cell proliferation in teleost fish, which provides a novel perspective on the understanding of the multiple function and evolutionary origins of C-type lectins in the immune system.

## Introduction

The immune system consisting of innate immunity and adaptive immunity is a complex organizational system, in which the body produces immune responses and performs immune functions. In this complex system, the immune cells are the important and indispensable part, and the structural integrity and normal function of immune cells are the cornerstone of maintaining the body’s immune homeostasis, such as monocytes (MO), macrophages (MФ), lymphocytes, dendritic cells (DCs), granulocytes, and mast cells. Among them, the MO/MФ as one of the most important innate immune cells, are distributed in almost all tissues, connecting the innate immunity and adaptive immunity, showing a great functional diversity (1, 2). The proliferation, differentiation, movement and apoptosis of the MO/MФ are the manifestation of the normal physiological functions, which are regulated by a series of biological events, enabling cells to survive and expand normally. The MO/MФ also rapidly produce and respond moderately to stimuli from the internal and external environment, thereby performing immune defense, surveillance and homeostatic functions (1, 3).

In normal organisms, the various biological activities of cells are closely related, such as proliferation, differentiation and apoptosis (4, 5). Cell proliferation, acting as one of the important processes in cell life, is the basis of growth, development, reproduction and heredity of organisms. Generally, proliferation and differentiation are the development of normal life activities of the body, and the cell cycle is the premise and basis of proliferation (6). While, apoptosis is a declining process of cell life, which plays an essential role in the elimination of abnormal and unnecessary cells (5, 7). Apoptosis and proliferation seem to be opposites, but they are closely related, inseparable in the life activities of normal organisms (4). As is known to all, the essence of the cell proliferation is a cyclic process of cell cycle, which is an orderly and fine regulation process involving multiple stags, factors and pathways, such as TGF-β1, MAPK and PI3K/AKT pathway (8–10). In the meanwhile, a growing body of studies points out that pattern recognition molecules, including TLRs and C-type lectins are also important in the regulation of the cell proliferation (11–14).

Mannose-binding lectin (MBL), a typical representative of C-type lectins, is a key pattern recognition molecule that plays an important role in the body’s immune defense. MBL is a secreted protein primarily synthesized by hepatocytes and widely present in various tissues (15, 16). Particularly, the MBL can not only recognize and bind various microorganisms, but also participate in downstream effector functions, such as agglutination, opsonophagocytosis, complement activation and killing, which plays multiple roles in the first line of host defense (17–20). In addition, the MBL can also interact with different types of host cells and play an important role in immune regulation (11, 12, 21, 22). Mammalian MBL has been reported to regulate dendritic cell maturation, and cytokine production upon the activation of bacterial lipopolysaccharide (LPS) (21, 22). In-depth studies also confirm that the MBL can regulate the expression of chemokine CXCL2 in hepatic macrophages (23). Furthermore, the MBL can also directly interact with T cells and MO, and affect the immune system by inhibiting the proliferation of these cells (11, 12).

Although the essential roles of MBL in innate immune defense and immune regulation by related signaling pathways have been extensively studied in mammalian models, the corresponding mechanism in early vertebrate remains an enigma. From an evolutionary viewpoint, fish, including cartilaginous and teleost fish, are important models for comparative immunology studies. In recent studies, the teleost MBL has been identified in many species, including common carp (*Cyprinus carpio*), rainbow trout (*Oncorhynchus mykiss*), channel catfish (*Ictalurus punctatus*), zebrafish (*Danio rerio*) and grass carp (*Ctenopharyngodon idella*) (15, 24–28). These studies mainly focus on the gene cloning, binding capacity, opsonophagocytosis, and complement activation of MBL; however, the studies on the mechanism of the MBL involved in immune regulation are rare limited, especially in the regulation of cell proliferation. We previously identified and purified the MBL from Nile tilapia (*Oreochromis niloticus*) (OnMBL), and pointed out that the OnMBL was able to agglutinate bacterial pathogens and participate in the regulation of non-specific cell immunity (16, 20, 29). These results indicate that the teleost MBL has already evolved sophisticated regulatory strategies for relevant immunobiological processes. However, almost all existing studies in fish have focused on their defensive function in innate immunity (15, 24–29). Whether and how they play a role in immune regulation of fish remains unclear. Combined with previous research results, further exploration on the relevant regulatory function and mechanism of the OnMBL are of great interest to us. Therefore, in this study, using Nile tilapia as a model, we investigated the effect and regulatory mechanisms of OnMBL on MФ proliferation. Our results indicated that the OnMBL is likely to participate in the regulation of MФ proliferation through the classic TGF-β1/Smad signaling pathway, thereby maintaining cellular homeostasis in the body’s internal environment. To our knowledge, these results provide new insights into the understanding of the MBL as a regulator of cell proliferation in a primary animal.

## Materials and methods

### Animals

Nile tilapia, about 300 ± 20 g were obtained from Guangdong Tilapia Breeding Farm (Guangzhou, China). Prior to experiments, fish were acclimated in a recirculating water system under 28 ± 2°C for 2 weeks, as previously described (16, 30). All animal protocols were reviewed and approved by the University Animal Care and Use Committee of the South China Normal University (an approval reference number SCNU-SLS-2021-005).

### Isolation and culture of macrophages

The separation of Nile tilapia head kidney MФ was performed according to the previous methods with some modifications (26, 29, 31). Briefly, the cells from head kidney were isolated through a 54%/31% discontinuous percoll (Sigma, USA) density gradient. Then, the cells at the junction were collected and adjusted to 2 × 10^7^ cells/mL with L-15 medium (Gibco, USA) including 10% fetal bovine serum and 1% penicillin/streptomycin. Following a 72 h incubation at 25°C, the non-adherent cells were removed. Ultimately, the attached MФ were collected and resuspended in L-15 medium to 1 × 10^6^ cells/mL after washing three times.

### Analysis of OnMBL binding to macrophages

The OnMBL eukaryotic protein (endotoxin-removed) and mouse against OnMBL polyclonal antibodies (pAb) have been obtained in our previous study (29). For eukaryotic protein, the plasmid of *OnMBL* was inserted into the expression vector pcDNA3.1 with N-terminal His tag, which were transfected into the Chinese hamster ovary cells and purified affinity chromatography on Ni-NTA Sepharose columns as described previously (16, 29). The MФ (1 × 10^6^ cells/mL) were resuspended in TBS-Ca^2+^ buffer (10 mM TBS, 2 mM CaCl_2_), pH 7.4. The cell suspension (200 μL) was incubated for 1 h at 25°C in the presence of TBS, BSA (5 μg/mL) or OnMBL (5 μg/mL), respectively, which had been previously labeled with fluorescein isothiocyanate (FITC; Sigma, USA). After washing, the binding of OnMBL to MФ was analyzed by flow cytometer (BD, USA).

In order to verify the binding of OnMBL to MФ, immunocytochemistry and western blot were also performed. Briefly, the MФ (1 × 10^6^ cells/mL) were incubated with TBS or OnMBL (5 μg/mL) for 1 h at 25°C. After washing three times, the cells were fixed by 4% paraformaldehyde for 10 min and blocked with 3% bovine serum albumin (diluted in TBS), then incubated with mouse anti-tilapia OnMBL pAb (1:1000, 1 μg/mL) for 3 h at 25°C. Subsequently, the cells were incubated with goat anti-mouse IgG monoclonal././././Program Files (x86)/Youdao/Dict/7.1.0.0421/resultui/dict/?keyword=antibody (Ab) conjugated with Alexa Fluor 488 (1:2000, 0.5 μg/mL) (Thermo, USA) for 1 h. The cell nuclei were stained with 4-6-diamidino-2-phenylindole (DAPI) for 10 min. After washing four times with TBS, the slides were observed by using a fluorescence microscope (Leica). For western blot analysis, the MФ (1 × 10^6^ cells/mL) were incubated with TBS or OnMBL (5 μg/mL) for 1 h at 25°C. After at least four washes, the samples were separated by 12% SDS-PAGE, and then the protein was transferred to nitrocellulose membrane at 100 V for 1 h. After blocking with 3% bovine serum albumin (diluted in TBS), at room temperature for 1 h, the blots were incubated with mouse anti-tilapia OnMBL pAb (1:1000, 1 μg/mL) for 1 h. After washing three times, the blots were further incubated with 1:20,000 diluted goat anti-mouse IgG conjugated with Alexa Fluor 680 (Southern Biotech, USA) as the secondary antibody for 1 h. Another washing three times, the blotting results were acquired by using Odyssey CLx Image Studio.

The binding of OnMBL and MФ was detected by ELISA. Briefly, the 96-well plates were coated with 100 μL MФ (1 × 10^7^ cells/mL) and centrifuged at 500 × *g* at 4°C for 5 min. Then, the cells were fixed by 4% paraformaldehyde for 30 min. After washing, the cells were blocked with 0.5% bovine serum albumin at 37°C for 2 h. One hundred microliters of OnMBL, BSA and Trx-pET-32a protein (Trx) (16) with different concentrations were added to the corresponding wells for 1 h. Among then, the OnMBL and Trx protein were pre-labeled with biotin hydrazide (Sigma-Aldrich) following the manufacturer’s instruction, respectively. The binding effect was detected by streptavidin–HRP conjugate (1:2500; SouthernBiotech). In addition, the cells were incubated in the presence of TBS, OnMBL (5 μg), OnMBL + OnMBL pAb (1:1500), or BSA (5 μg). Meanwhile, 2 mM CaCl_2_ was added into the buffer. The primary antibody was mouse against OnMBL pAb (1:1500). The secondary antibody was horseradish peroxidase-conjugated goat anti-mouse IgG Ab (1:2000; Southern Biotech, USA). After adding the tetramethylbenzidine (TMB) substrates, the reaction was terminated by 1 M H_2_SO_4_ and the absorbance was read at a wavelength of 450 nm. The absorbance represented the enzyme-substrate color change. The ELISA assay was employed by referenced to the previous methods (16, 32). Moreover, *Streptococcus agalactiae* (1 × 10^7^ CFU/mL, formalin-inactivated), *Aeromonas hydrophila* (1 × 10^7^ CFU/mL, formalin-inactivated), LPS (100 ng/mL), LTA (100 ng/mL) and TBS (control) were pre-incubated with MФ for 2 h, respectively. Then, the binding of pre-incubated MФ to OnMBL (5 μg/mL) was also detected as above.

### Proliferation assays

To investigate the effect of OnMBL in MФ proliferation, the flow cytometric analysis was performed. Detailed steps were performed according to the protocols of BeyoClickTM Edu Cell Proliferation Kit with Alexa Fluor 488 (Beyotime, China). Briefly, human macrophage colony stimulating factor (50 ng/mL; hM-CSF; Rocky Hill, USA)-induced MФ (5 × 10^5^ cells/mL, 50 μL) were cultivated into 96-well plates. Then, different concentrations of OnMBL (5 μg/mL and 50 μg/mL) were added and the plates (100 μL/well) were incubated at 25°C for 24, 48 and 72 h. Among them, the selection of OnMBL concentration was mainly referred to previous studies (11, 20). In addition, the BSA (50 μg/mL) and TBS group were served as control. Every 24 hours, the cells were labeled by using the Edu (10 μM) and incubated 2 h. Subsequently, the cells were fixed for 15 min and permeabilized for 10 min. After washing, Click reaction solution (50 μL) was added to each well and incubated in the dark for 30 min. After washing, flow cytometry analysis was performed.

The Cell Counting Kit-8 (CCK-8) assay was also used to detect the effect of OnMBL on MФ proliferation. The MФ (5 × 10^5^ cells/mL) were incubated in 96-well plates and the hM-CSF was added to the concentration of 50 ng/mL. Different concentrations of OnMBL (5 μg/mL and 50 μg/mL) were also added and the plates were incubated at 25°C for 24, 48 and 72 h. The BSA (50 μg/mL) and TBS group were served as control. Every 24 hours, CCK-8 solution (10 μL; AbMole, USA) was added to each well and incubated in the dark for 2-4 h. The absorbance was read at a wavelength of 450 nm by Microplate Reader (Thermo, USA). In addition, the CCK-8 assay was also used to detect the effect of OnMBL on the proliferation of *M. musculus* peritoneal MФ (J774A.1). The specific method was described as above. Moreover, the expression levels of MФ proliferation markers *Ki-67* and *Maf* were detected by quantitative real-time PCR (qRT-PCR) after OnMBL pre-incubation for 12, 24, 48 and 72 h.

### RNA isolation and qRT-PCR analysis

Total RNA was extracted by using Trizol Reagent (Invitrogen, USA) according to the manufacturer’s instructions. The quantification of the extracted RNA was measured by using NanoDrop 2000 spectrophotometer, and the integrity of the RNA was determined by 1% agarose gel electrophoresis (BIOWEST, Spain). Equivalent amount of the total RNA (1000 ng) from each sample was used for cDNA synthesis with PrimerScript™ RT reagent kit with gDNA Eraser (TaKaRa, Japan) for qPCR (Bio-Rad, USA) in a 20 μL reaction volume. The prepared cDNA templates were store at -20°C, and a 10-fold dilution was required in qRT-PCR. The qRT-PCR was performed on a 7500 Real-time PCR system (Life Technologies, USA). The PCR reaction volume was 20 μL, containing 10 μL 2 × TaKaRa Ex Taq™SYBR premix, 3 μL of diluted cDNA, 2 μL of each primer (2 μM), 2.6 μL DEPC treated water (Invitrogen, USA), 0.4 μL Rox Reference Dye II (Takara, Japan). The PCR program was 95°C for 3 min, 95°C for 15 s, 60°C for 1 min then go to step 2 and repeated for 40 cycles. CT values determined for each sample were normalized against the values for housekeeping gene *β*-actin. The results were further compared to respective control group to determine the change of gene expression by the 2^−ΔΔCt^ method (33). The primers used for qRT-PCR were listed in **Supplemental Table 1**.

### Cell cycle analysis

Flow cytometric analysis was performed to explore the effect of OnMBL on the cell cycle of MФ. Briefly, the pre-prepared MФ (5 × 10^5^ cells/mL) were treated with OnMBL (5 μg/mL or 50 μg/mL) for 24, 48, and 72 h, respectively. The cells were collected and fixed in 70% alcohol at 4°C overnight. Then, cells were stained with 500 μL propidium iodide (PI) staining buffer (100 μg/mL RNase A, 50 μg/mL PI; Beyotime, China) for 30 min at 37°C in the dark. Cell cycle distributions were analyzed by flow cytometry. Moreover, the expression levels of cyclin (*Cyclin D1*, *Cyclin D2*, *Cyclin E2*), cyclin dependent kinase (*CDK2*, *CDK4*, *CDK6*), and cyclin dependent kinase inhibitor (*p21*, *p27*, *CDKN3*) were detected by qRT-PCR after OnMBL pre-incubation for different times.

### Apoptosis analysis

The pre-prepared MФ (5 × 10^5^ cells/mL) were treated with OnMBL (5 μg/mL or 50 μg/mL), BSA (50 μg/mL) and TBS for 24, 48, and 72 h, respectively. All the groups were cultured at 25°C. Apoptosis experiment was performed by flow cytometry analysis according to the manufacturer’s instructions from FITC-Annexin V apoptosis detection kit I (BD, USA). The cells were collected and re-suspend in Annexin V-FITC binding buffer (190 μL). Subsequently, five microliter of FITC-Annexin V and 5 μL PI were added to the cell suspension, then the mixture was incubated in dark for 15 min before flow cytometer analysis.

At the same time, the apoptosis of treated MФ was also observed by laser confocal microscopy (Zeiss, Germany). The assay was performed by one step TUNEL (TdT-mediated FITC-dUTP Nick-end Labeling) apoptosis assay kit (Beyotime, China). Briefly, 50 μL TUNEL detection solution was added to the treated-MФ, then the mixture were incubated in dark for 60 min. After washing, the cells were incubated with 0.5 μg/mL of DAPI (Sigma, USA) for 10 min. Then the cells were washed again and subjected to microscopy observation. Moreover, the expression levels of apoptosis-related regulatory molecules (*Bax*, *Bcl-2*, *FasL*, *FAIM*, and *Caspase-3*) in the MФ were detected by qRT-PCR after OnMBL pre-incubation.

### Sequence, structure, and phylogenic analysis

All sequences were acquired from National Center for Biotechnology Information (https://www.ncbi.nlm.nih.gov), and analyzed by using the BLAST algorithm. The functional domains of proteins were predicted with the simple modular architecture research tool (SMART) version 4.0, and sketch maps were drawn by using DOG version 2.0. Multiple sequence alignments were generated by using ClustalW Multiple Alignment program based on homology analysis (http://www.bioinformatics.org/sms/), and dimensional protein structure was predicted by AlphaFold 2.0. Neighbor-joining trees were constructed by using MEGA version 6.0 with 1,000 bootstrap replications. All of the primers used in this study were listed in the **Supplemental Table 1**.

### TGF-β1 preparation

The ORF of *OnTGF-β1* was cloned based on the sequence of *Oreochromis niloticus TGF-β1 mRNA* (GenBank accession XP_025753606.1). The PCR products were test by a 1% agarose gel electrophoresis. The expression primers were designed with restriction sites (*EcoR1* and *Hind III*), ETGF-β1-F and ETGF-β1-R (**Supplemental Table 1**). The expression plasmid Trx-pET-32a-TGF-β1 was obtained and transformed into *E.coli* BL21 (DE3) (TianGen, China), and cultured in LB liquid media with ampicillin (100 μg/mL) at 37°C. The (r)OnTGF-β1 was expressed and purified as the previous description (16). The purification of (r)OnTGF-β1 was referred to the manufacturer’s protocols by the His Band Resin columns (Novagen, Germany). The purified (r)OnTGF-β1 was detected by a 12% SDS-PAGE gel electrophoresis and western blot analyses. The primary antibody was rabbit anti-human TGF-β1 pAb (1:1500; Affinity Biosciences, USA) or anti-His Ab (1:1500; BBI Life Sciences, USA).

### Detection of TGF-β1 pathway-related molecules

The MФ were prepared and adjusted to 1 × 10^6^ cells/mL with fresh L-15 medium as above description. The treatment group was challenged with OnMBL (5 μg/mL or 50 μg/mL), or BSA (5 μg/mL or 50 μg/mL), and the control group was treated with an equal volume of TBS. All groups were maintained at 25°C, and cells were collected and lysed with Trizol Reagent for RNA extraction at the time of 12, 24, 48 and 72 h post-challenge. Moreover, the expression levels of TGF-β1 pathway-related molecules including *TGF-β1*, *TGFBR1*, *TGFBR2*, *Smad2*, *Smad3*, and *Smad4* in the MФ were also detected by qRT-PCR.

### Measurement of OnTGF-β1 by ELISA

The OnTGF-β1 concentration in the supernatant of MФ was tested by a competitive inhibition ELISA as reported previously (34). Briefly, the 96-well plates were coated with (r)OnTGF-β1 (2 μg/mL), diluted with a coating buffer (0.05 M carbonate-bicarbonate buffer, pH 9.6) at 4°C overnight. The plates were then blocked with 0.5% BSA-TTBS for 2 h at 37°C. The stimulated culture supernatant and rabbit anti-human TGF-β1 pAb (1:2,000, the optimal dilution determined previously) were placed in each well and incubated for 1 h. The second antibody was HRP-conjugated goat anti-rabbit IgG Ab (1:2,000; Southern Biotech, USA). Finally, the result was detected by a microplate reader (Thermo, USA) at O.D. 405 and calculated from pre-made standard curves.

### Application analysis of TGF-β1 receptor inhibitor

The hM-CSF (50 ng/mL) induced-MФ were incubated with TGF-β1 receptor inhibitor SB-431542 (5 μM), anti-TGF-β1 pAb (0.1 μg/mL), or rabbit IgG (0.1 μg/mL) for 1 h. Then, the OnMBL (50 μg/mL) was added and incubated at 25°C for 24, 48, and 72 h, respectively. The proliferation of MФ in each group was assessed by the CCK-8 assay as above. Moreover, the expression levels of *TGF-β1*, *TGFBR1*, *TGFBR2*, *Smad2*, *Smad3*, and *Smad4* in MФ were detected by qRT-PCR after OnMBL with or without SB-431542 pre-incubation for 72 h.

### Immunofluorescence and western blot analysis

The MФ with or without OnMBL stimulation were washed and fixed for 15 min. After blocked with 3% BSA-TTBS at room temperature for 1 h, the cells were then incubated with rabbit anti-human TGFBR1 pAb (1:500; Affinity Biosciences, USA), rabbit anti-human Smad2/3 pAb (1:500; Affinity Biosciences, USA), or rabbit anti-human phospho-Smad2/3 pAb (1:500; Affinity Biosciences, USA) for 3 h at room temperature. Subsequently, the samples were washed and then incubated with donkey anti-rabbit IgG Ab conjugated with Alexa Fluor 594 (1:1000; Thermo, USA) for 1 h. The cell nuclei were stained with DAPI for 10 min. After washing, the samples were observed under the laser confocal microscope.

Moreover, the protein expression or phosphorylation levels of the indicated TGF-β1 pathway components in the stimulation MФ with or without SB-431542 were also detected by western blot analysis. The primary antibodies were rabbit anti-human TGFBR1 pAb (1:1000), rabbit anti-human Smad2/3 pAb (1:1000), or rabbit anti-human phospho-Smad2/3 pAb (1:1000), respectively. The second antibody was goat anti-rabbit IgG Ab (1:2000).

### Statistical analysis

All of the experiments were performed at least three times and statistical analyses were carried out with SPSS 17.0 software. Data are presented as the mean ± standard deviation, and statistical significance was determined with analysis of variance (ANOVA) followed by two-tailed Student’s *t* test. The *p* values are defined by different letters (a, b, c, and d) (*p* < 0.05) or asterisks (*p < 0.05, **p < 0.01). The figures in this study were made by Sigma Plot 10.0 software.

## Results

### OnMBL binds to MФ

**Figure 1:**
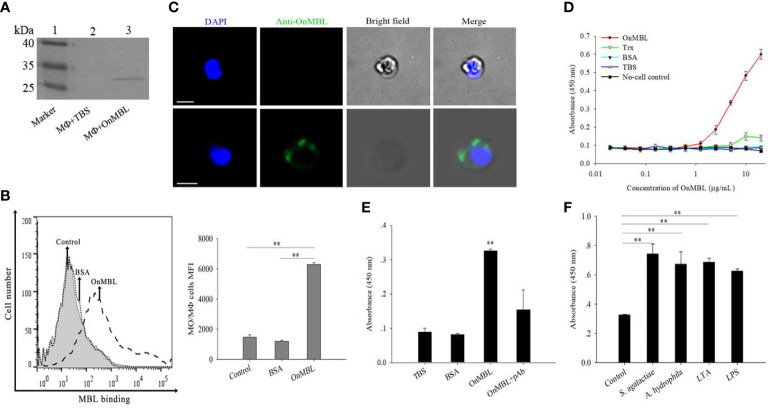
Detection of binding of OnMBL to MФ. The binding of OnMBL (5 μg/mL) with MФ was detected by western blot **(A)**, flow cytometry **(B)**, and immunofluorescence **(C)** (mouse anti-OnMBL pAb as the primary Ab). **(D)** The binding of MФ to OnMBL at different concentrations was detected by ELISA. **(E)** The effects of mouse anti-OnMBL pAb (10 μg) on OnMBL (5 μg/mL) binding to MФ was detected. **(F)** Binding analysis of MФ to OnMBL pre-incubated with *S. agalactiae* (1 × 10^5^ CFU/mL), *A. hydrophila* (1 × 10^5^ CFU/mL), LPS (10 μg/mL), or LTA (10 μg/mL), respectively. The error bars represent SD (n=4) and significant difference is indicated by asterisks (**p < 0.01).

Flow cytometry result showed that OnMBL protein (**Figure S1A**) could directly bind to MФ (**Figure 1B**), and the western blot and immunofluorescence analyses also showed consistent results (**Figures 1A, C**). The results of ELISA assay showed that OnMBL bound to the MФ with a dose-dependent manner, and the BSA control protein had no binding ability to MФ (**Figures 1D, E**). Interestingly, the MФ stimulated by bacteria (*S. agalactiae* and *A. hydrophila*) and polysaccharides (LPS and LTA) showed a stronger ability to combine with OnMBL (**Figure 1F**).

### OnMBL regulates proliferation of MФ

**Figure 2:**
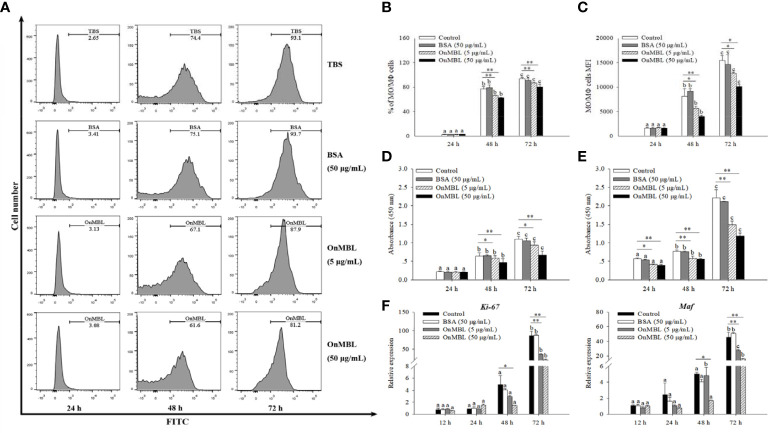
OnMBL regulates MФ proliferation. **(A)** Effects of OnMBL on proliferation of MФ stimulated with 50 ng/mL hM-CSF were assessed by the BeyoClick™ EdU Cell Proliferation Kit with Alexa Fluor 488 assay. Flow cytometric histogram analysis of the effect of OnMBL on MФ proliferation at different time points. The total average percentage **(B)** and the mean fluorescence intensity (MFI, **C**) of MФ. Effects of OnMBL on proliferation of *O. niloticus* MФ **(D)** and *M. musculus* peritoneal MФ **(E)** stimulated with 50 ng/mL hM-CSF were assessed by the CCK-8 assay. **(F)** The effect of OnMBL on the mRNA expressions of cell proliferation marker molecule *Ki-67* and *Maf* was analyzed by qRT-PCR. The error bars represent SD (n=4) and significant difference is indicated by different letters (a–c) (*p* < 0.05) or asterisks (*p < 0.05, **p < 0.01).

To explore the correlation between the proliferation of MФ and level of OnMBL, a series of experiments were performed. Flow cytometry results showed that OnMBL (5 μg/mL and 50 μg/mL) had a significant inhibitory effect on the proliferation of hM-CSF-activated MФ for 48 h and 72 h in a concentration-dependent manner (**Figures 2A–C**). However, the BSA group had no inhibitory effect on the cell proliferation compared with the control group. In addition, the results of CCK-8 assay showed that the same concentration of OnMBL could significantly decrease the proliferation of hM-CSF-activated MФ at 48 h and 72 h (**Figure 2D**; **Figure S1B**). Meanwhile, the CCK-8 assay confirmed that OnMBL could also significantly reduce the proliferation of *M. musculus* peritoneal MФ (**Figure 2E**). After challenged with OnMBL (5 μg/mL and 50 μg/mL), the expressions of the MФ proliferation marker molecules *Ki67* and *Maf* were significantly down-regulated when compared with the control group (**Figure 2F**). Visibly, OnMBL could regulate the proliferation of activated-MФ in a time- and concentration-dependent manner, indicating that the proliferative response of MФ was closely correlated with the level of MBL.

### OnMBL induces cell arrest in the G0/G1 phase of MФ cell cycle

**Figure 3:**
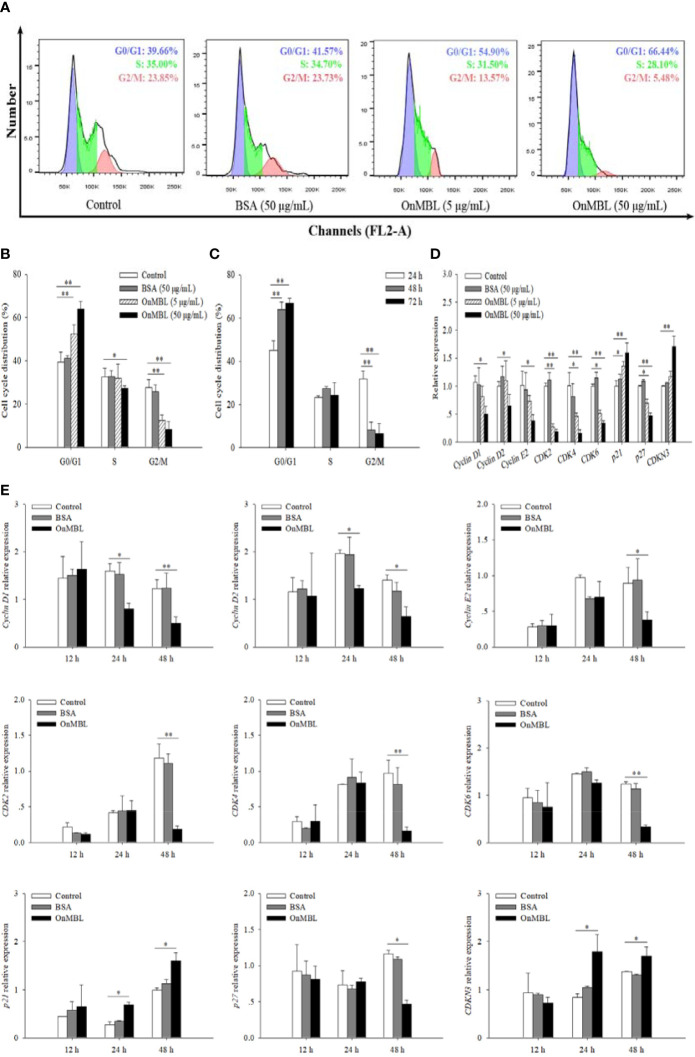
OnMBL causes a G0/G1 arrest in cell cycle of MФ. **(A)** The MФ were stimulated with hM-CSF (50 ng/mL) in the presence or absence of OnMBL (5 μg/mL, 50 μg/mL) for 72 h. Cells were harvested and stained with PI, followed by flow cytometric analysis. **(B)** Summaries of four independent experiments were shown. **(C)** Effects of OnMBL (50 μg/mL) on cell cycle of MФ at 24 h, 48 h and 72 h post-challenge. **(D)** The effect of different concentrations of OnMBL on the mRNA expression of cell cycle regulatory molecules was analyzed at 48 h post-challenge by qRT-PCR. **(E)** Effects of OnMBL (50 μg/mL) on cell cycle regulatory molecules of MФ at 12 h, 24 h and 48 h post-challenge. The average SD was obtained from three independent experiments. *p < 0.05, **p < 0.01, difference from control.

To further investigate the effect of OnMBL on MФ cell cycle, flow cytometry analysis was performed. Representative flow histograms depicting cell cycle distribution showed that more MФ were remained in G0/G1 phase after OnMBL stimulation compared with the control group, and accompanied by a decrease in G2/M phase (**Figure 3A**). With the increase of OnMBL concentration, the proportion of cells in the G0/G1 phase increased significantly (**Figures 3A, B**). The results indicated that the OnMBL might induce cell arrest in the G0/G1 phase of the MФ cell cycle. After treated with OnMBL (50 μg/mL) for 24, 48, and 72 h, the proportion of the MФ in the G0/G1 phase increased and the proportion of cells in the G2/M phase decreased, and this was positively with the incubation time (**Figure 3C**). Moreover, the expression levels of cyclins and cyclin dependent kinases such as *Cyclin D1*, *Cyclin D2*, *Cyclin E2, CDK2*, *CDK4* and *CDK6* were significantly down-regulated in a concentration- and time-dependent manner after challenged by OnMBL (**Figures 3D, E**). However, the expression levels of cyclin dependent kinase inhibitor including *p21* and *CDKN3* were significantly up-regulated after OnMBL stimulation (**Figures 3D, E**). Strangely, the expression level of cyclin dependent kinase inhibitor *p27* was also significantly down-regulated after OnMBL challenge (**Figures 3D, E**). These results further confirmed that OnMBL reduced MФ proliferation by arresting a large number of cells in the G0/G1 phase of cell cycle.

### OnMBL induces apoptosis

**Figure 4:**
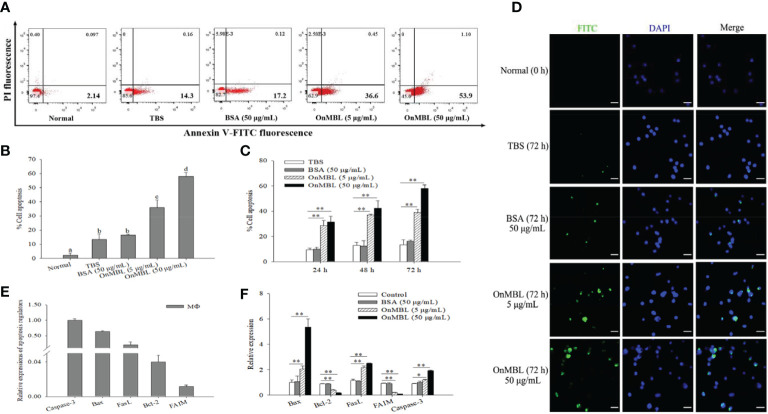
Induction of apoptosis of MФ following treatment with OnMBL. **(A)** The induction of apoptosis was determined by Annexin V-FITC/PI double-staining assay. MФ were treated with OnMBL (5 μg/mL or 50 μg/mL), BSA (50 μg/mL) or PBS for 72 h. **(B)** The histogram of the apoptosis rates of four independent experiments were summarized. **(C)** Effects of OnMBL on cell apoptosis of MФ at 24 h, 48 h and 72 h post-challenge were investigated. **(D)** The induction of apoptosis was detected by fluorescence microscopy. **(E)** The mRNA expression level of cell apoptosis regulatory molecules in MФ. **(F)** The effect of different concentrations of OnMBL on the mRNA expression of cell apoptosis regulatory molecules was analyzed at 48 h post-challenge by qRT-PCR. The error bars represent SD (n=4) and significant difference is indicated by different letters (a-c) (*p* < 0.05) or asterisks (*p < 0.05, **p < 0.01).

Generally, apoptosis is also closely related to proliferation, which plays a necessary role in eliminating unwanted or abnormal cells in the body. In order to explore the further effect of OnMBL on cell apoptosis, the apoptosis rate of MФ was performed by flow cytometer analysis. The results showed that OnMBL could significantly increase the level of apoptosis in MФ at 72 h compared with the control group (**Figures 4A, B**). The apoptosis rate of cells after treated with OnMBL (5 μg/mL or 50 μg/mL) raised significantly from 24 h to 72 h (**Figure 4C**). These results showed that OnMBL could induce apoptosis of MФ with a dose-and time-dependent manner. Further, immunofluorescence detection performed by TdT-mediated FITC-dUTP Nick-end labeling method also found that OnMBL could induce the apoptosis (green fluorescence) of MФ (**Figure 4D**). Moreover, the expression levels of apoptosis-related regulators including *Bax*, *FasL*, and *Caspase-3* were significantly up-regulated (**Figures 4E, F**); however, the expression levels of anti-apoptotic molecules *Bcl-2* and *FAIM* were significantly down-regulated after OnMBL challenge (**Figures 4E, F**). These results showed that OnMBL could induce apoptosis in a concentration- and time-dependent manner. This might be related to the phenomenon that OnMBL could induce a large number of cells to stagnate in G0/G1 phase and not enter S phase normally, thus increasing the risk of apoptosis.

### OnMBL induces secretion of TGF-β1 and reduces macrophage proliferation

**Figure 5:**
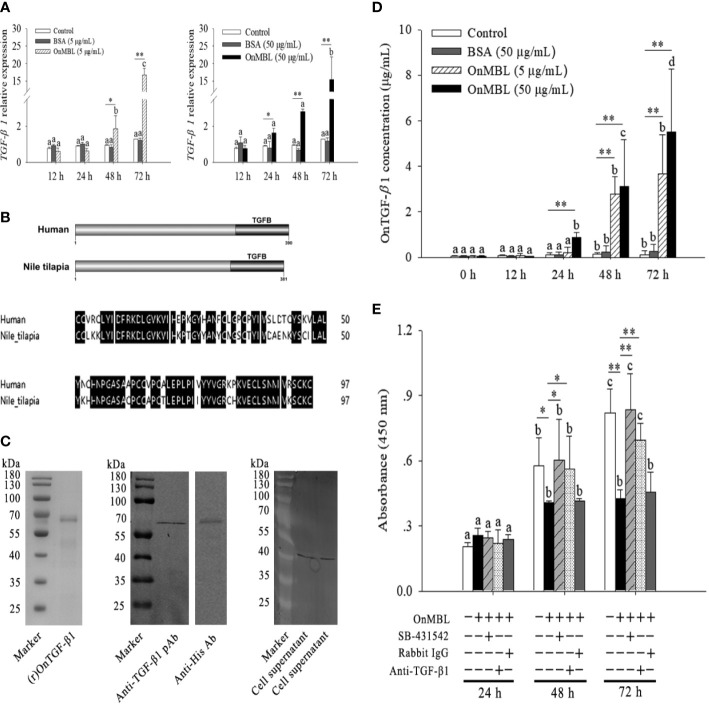
OnMBL inhibits MФ proliferation by TGF-β1. **(A)** Expression level of TGF-β1 in MФ was incubated with OnMBL at 5 μg/mL or 50 μg/mL. **(B)** The domain and multiple sequence alignment analysis of the OnTGF-β1 and human TGF-β1. **(C)** Purification and western blot analysis of (r)OnTGF-β1 (rabbit anti-human TGF-β1 polyclonal antibody or anti-His monoclonal antibody as the primary antibody). The native OnTGF-β1 in the cell supernatant was also detected by western blot. The supernatant was harvested from MФ *in vitro* culture at 48 h after OnMBL stimulation. **(D)** The protein concentration of OnTGF-β1 in the supernatant of MФ after OnMBL (5 μg/mL or 50 μg/mL) challenge by ELISA analysis. **(E)** The MФ induced by hM-CSF (50 ng/mL) were incubated with OnMBL (50 μg/mL) alone or in the presence of TGF-β1 receptor inhibitor (SB-431542), anti-TGF-β1 pAb, rabbit IgG. Effects of OnMBL on proliferation of MФ were assessed by the CCK-8 assay. The error bars represent SD (n=4) and significant difference is indicated by different letters (a-c) (*p* < 0.05) or asterisks (*p < 0.05, **p < 0.01).

Based on the above findings, OnMBL has been shown to reduce the proliferation of activated-MФ by regulating the cell cycle, arrest a large number of cells in the G0/G1 phase, and induce the apoptosis. Then, the potential signaling pathway activated by OnMBL was investigated. As shown in **Figure 5A**, the mRNA expression of tilapia *TGF-β1* in MФ was significantly up-regulated after OnMBL (5μg/mL or 50 μg/mL) challenge. Meanwhile, the amplified ORF of Nile tilapia *OnTGF-β1* was 1146 bp and encoded 381 amino acid residues (**Figure S1C**). Nile tilapia TGF-β1 has high similarity in functional domain with human homolog (**Figure 5B**). Both TGF-β1 proteins contain a TGFB domain in the C terminal, which is essential for their functions (**Figure 5B**). The ORF of *OnTGF-β1* was cloned into pET-32a vector, transformed into BL21 (DE3) competent cells, and the recombinant protein fused with His-tag was purified and analyzed by SDS-PAGE and western blot. As shown in **Figure 5C**, a band (~64 kDa) corresponding to OnTGF-β1-His fusion protein was clearly detected. To verify the specificity of pAb (rabbit anti-human TGF-β1 pAb), the (r)OnTGF-β1 was detected by western blot analysis. The result showed a specific positive band about 64 kDa (the middle of **Figure 5C**). In addition, the TGF-β1 native protein (~44 kDa) was also detected in the MФ supernatant at 48 h after OnMBL (5 μg/mL or 50 μg/mL) challenge (the right side of **Figure 5C**). Moreover, the concentration of OnTGF-β1 in the cell supernatant of MФ was also increased significantly after OnMBL stimulation (**Figure 5D**). The above results indicated that OnMBL could induce the massive expression and secretion of TGF-β1 in MФ.

Interestingly, the inhibitory effect of OnMBL was significantly abolished by the addition of anti-TGF-β1 pAb; however, the addition rabbit IgG could not show this effect (**Figure 5E**). In-depth study showed that treatment of activated-MФ with the TGF-β1 receptor antagonist (SB-431542) could also abolish the inhibitory effect of OnMBL (**Figure 5E**). These results indicated that the function of OnMBL to reduce cell proliferation was closely related to the TGF-β1 pathway.

### OnMBL activates TGF-β1 signaling pathway to regulate MФ proliferation

**Figure 6:**
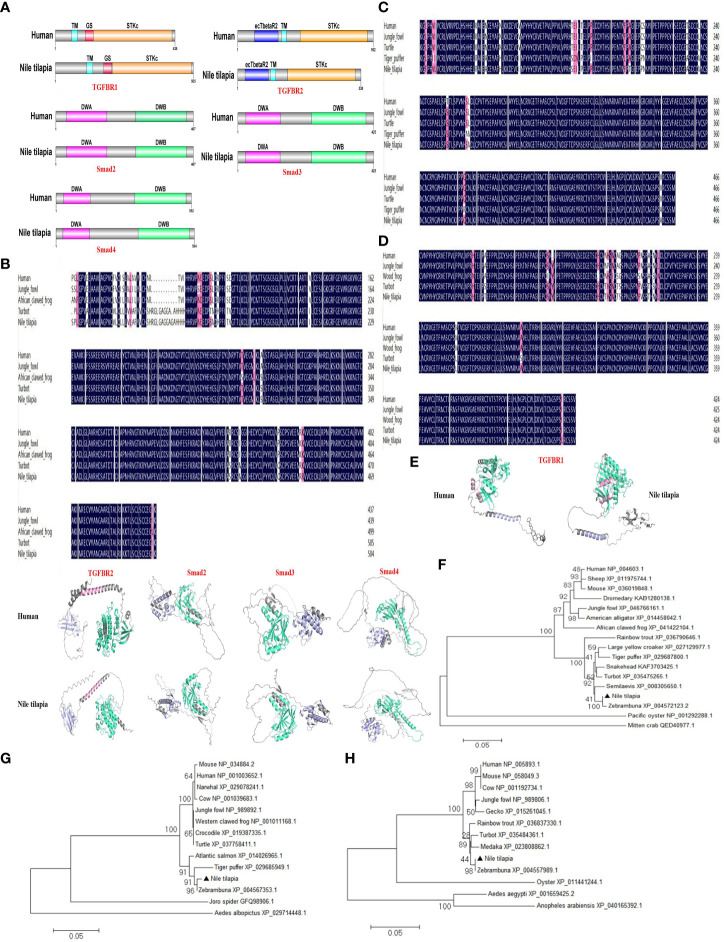
Evolutionary conservation of canonical TGF-β1 signaling pathway in Nile tilapia. **(A)** Comparison of the domain organization of the canonical TGF-β1 pathway components from the indicated species. **(B-D)** Multiple sequence alignment analysis of the TGFBR1 **(B)**, Smad2 **(C)** and Smad3 **(D)** from Nile tilapia with homologs in other animals. **(E)** Prediction of tertiary structures of the indicated canonical TGF-β1 pathway components from Nile tilapia and human by AlphaFold 2.0. **(F-H)** Phylogenetic trees constructed with the amino acid sequences of TGFBR1 **(F)**, Smad2 **(G)** and Smad3 **(H)** from the indicated species. The tree was constructed using the neighbor-joining (NJ) method with MEGA 6.0 program based on the multiple sequence alignment by Clustal W. Numbers at each branch indicated the percentage bootstrap values on 1,000 replicates.

To further explore the relevant molecular mechanisms, the components of TGF-β1 signaling pathway were searched in the Nile tilapia genome sequence available in NCBI GenBank database. Key components of the TGF-β1 pathway, including TGFBR1, TGFBR2, Smad2, Smad3, and Smad4 (**Figure 6**; **Figure S1D**), suggest the presence of a canonical TGF-β1 signaling pathway in this early vertebrate. As two key receptors of TGF-β1, Nile tilapia TGFBR1 and TGFBR2 have high similarity in functional domain organization with its human homolog. Both TGFBR1 and TGFBR2 proteins contain a Serine/Threonine protein kinase catalytic domain (STKc) in the C terminal (**Figure 6A**). In Nile tilapia, the three downstream signal transduction molecules of TGF-β1 receptors, including Smad2, Smad3, and Smad4, adopt the same strategy to arrange their functional domains, including a DWA domain in the N-terminal and a DWB domain in the C-terminal (**Figure 6A**). Multiple-sequence alignment found that the amino acid sequences of TGF-β1 pathway molecules including TGFBR1, Smad2, and Smad3 had highly similar to those of homologs from other species (**Figures 6B–D**). In addition, the components of canonical TGF-β1 pathway in the Nile tilapia and human homologs shared high similarity to tertiary structure (**Figure 6E**). Moreover, phylogenetic tree analysis showed that Nile tilapia TGFBR1, Smad2, and Smad3 distinctly classified into a cluster with their homologs in other teleost fish and displayed close evolutionary relationships (**Figures 6F–H**). Altogether, the results indicated that Nile tilapia possessed an intact and evolutionarily conserved canonical TGF-β1 pathway, which might perform regulatory functions similar to those in higher vertebrates.

**Figure 7:**
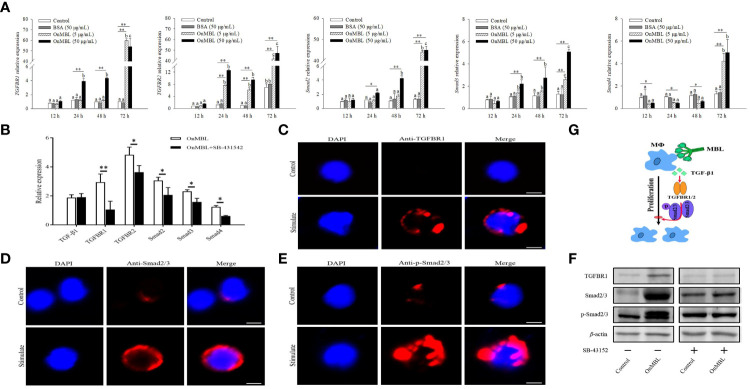
Effects of OnMBL on the TGF-β1 signaling pathway in MФ. **(A)** The effect of OnMBL (5 μg/mL or 50 μg/mL) on the mRNA expression of TGF-β1 signaling pathway-related molecules was analyzed at different time points by qRT-PCR. **(B)** The effect of OnMBL with or without SB-431542 on the expression levels of the TGF-β1 signaling pathway-related molecules in MФ. **(C-E)** Immunofluorescence analysis showing protein or phosphorylation levels of TGFBR1 **(C)**, Smad2/3 **(D)**, and p-Smad2/3 **(E)** with or without stimulation. **(F)** Western blot analysis showing protein or phosphorylation levels of the indicated TGF-β1 pathway components in MФ with or without stimulation. **(G)** Diagrammatic representation suggests how OnMBL regulates the TGF-β1/Smad pathway-related molecules to influence MФ proliferation. The error bars represent SD (n=4) and significant difference is indicated by different letters (a-c) (*p* < 0.05) or asterisks (*p < 0.05, **p < 0.01).

Next, we investigated how the TGF-β1 signaling pathway participates in OnMBL-mediated proliferation of MФ in Nile tilapia. Upon MФ activation, the expression levels of *TGFBR1*, *TGFBR2*, *Smad2*, *Smad3*, and *Smad4* were significantly up-regulated after the stimulation of OnMBL (**Figure 7A**). When SB-431542 was additionally added, the expression levels of *TGFBR1*, *TGFBR2*, *Smad2*, *Smad3*, and *Smad4* were significantly reduced compared with the OnMBL group (**Figure 7B**). Meanwhile, immunofluorescence detection also found that OnMBL could induce the large expression of TGFBR1 and Smad2/3 of MФ (**Figures 7C, D**). Additionally, increased phosphorylation of Smad2/3 upon MФ activation was also confirmed by fluorescence microscopy at the cellular level (**Figure 7E**). To further evaluate the regulatory role of the TGF-β1 signaling in the response of OnMBL to inhibiting cell proliferation, the expression and phosphorylation of TGF-β1 pathway-related molecules were detected by western blot analysis after co-incubating cells with SB-431542. As shown in **Figure 7F**, OnMBL could induce the expression of TGFBR1 and Smad2/3 in the activated-MФ, and also increase the phosphorylation of Smad2/3. However, the inducted effect of OnMBL was significantly abolished by the addition of SB-431542 (**Figure 7F**; **Figure S1E**). Therefore, the results, as shown in **Figure 7G**, suggested that OnMBL activated the TGF-β1 signaling pathway to regulate MФ proliferation in Nile tilapia.

## Discussion

MBL, as an importance member of C-type lectins, is originally described to activate complement system and defend infection. With the gradual deepening of research, MBL, as a bridge linking innate and adaptive immunity, can not only mediate the recognition of pathogenic microorganisms and their products, playing a pleiotropic role in innate immune defense, but also participate in adaptive immune responses (11, 22, 29). At present, understanding of the immune mechanisms of MBL remains limited, especially in teleost fish. In this study, we explored the regulatory function and mechanism of OnMBL on the proliferation of Nile tilapia MФ. The OnMBL reduces the proliferation of activated-MФ by regulating the cell cycle, arresting a large number of cells in the G0/G1 phase, and inducing apoptosis, which is achieved by activating the TGF-β1 signaling pathway, thereby maintaining the body’s cellular homeostasis.

Cell proliferation is one of the important physiological functions of living cells and an important life feature of organisms, which is the basis of organism growth, development, reproduction, and genetic inheritance. In mammals, MBL can interact with different types of host cells and play a regulatory role in cell proliferation, differentiation and apoptosis. For example, MBL regulates dendritic cell maturation and cytokine production induced by lipopolysaccharide (21, 22). MBL also directly interacts with T cells and suppresses T cell proliferation (11). In this study, we found that OnMBL could also effectively reduce the proliferation of activated-MФ. Generally, cell proliferation is an orderly regulatory process, which is achieved through the cell cycle. The essence of cell proliferation is the continuous cycle of cell cycle. The cell cycle is generally divided into G1 phase (prophase DNA synthesis), S phase (DNA synthesis phase), G2 phase (late DNA synthesis phase) and M phase (mitosis phase) (35, 36). Among them, the G1 phase is an important period for regulating the cell cycle. Once it enters the S phase through the G1 phase, the cells will no longer rely on the stimulation of exogenous proliferation and division signals to complete the cell cycle autonomously (35, 37). Research found that OnMBL could arrest cell cycle in G0/G1 phase, by hindering a large number of cells to stay in the early stage of DNA replication, and not entering the S phase. The proliferation of cells is precisely controlled by the cell cycle, and the normal operation of the cell cycle requires the coordinated control of many factors such as cyclins, cyclin dependent kinase, and cyclin dependent kinase inhibitor (38, 39). In mammals, the binding of cyclin D to the kinases CDK4 or CDK6, and the binding of cyclin E to CDK2 are key factors in regulating the progression of the G1/S phase of the cell cycle (39). Meanwhile, p21 and CDKN3 are negative regulatory proteins, which are the main inhibitory factors of cells from G1 phase to S phase (38, 40, 41). In this study, we also found that OnMBL could up-regulate the expression of *p21* and *CDNK3*, down-regulate the expression of *cyclin D*, *cyclin E*, *CDK2*, *CDK4*, and *CDK6*, block cells from entering S phase, and arrest a large number of cells in G0/G1 phase. These results provide strong support for that OnMBL suppresses MФ proliferation by regulating the cell cycle.

OnMBL induced cell cycle of MФ arrest in G0/G1 phase, which led to the cells cannot enter the cycle growth normally. Whether these cells stuck in the G0/G1 phase would increase the probability of apoptosis. Apoptosis is also the basic process of cell life activities. Under certain physiological, pathological or environmental conditions, the body regulates the balance between the proliferation and cell death, and causes the cells to die actively and orderly through genes control, thereby maintaining the stability of the internal environment (4). Apoptosis is a process of strict control of multiple genes, which are conserved among species, such as Bcl-2 family, Caspase family and Fas/Fasl gene (7, 42, 43). Study found that OnMBL could up-regulate the expressions of *Bax*, *FasL* and *Caspase-3*, and down-regulate the expressions of *Bcl-2* and *FAIM*, thereby inducing apoptosis of MФ. These findings were similar to that of study on human MBL in monocytes (12). Visibly, OnMBL reduces the proliferation of activated-MФ by regulating the cell cycle, arresting a large number of cells in the G0/G1 phase, and increasing the proportion of apoptosis, thereby maintaining internal environment stability.

TGF-β1 is one of the most ancient transcription growth factors and multifunctional cytokines found in invertebrates and vertebrates (10, 44–47). TGF-β1 signaling pathway is involved in many cellular processes and plays important regulatory functions in both developing embryos and mature organisms, including cell growth, proliferation, differentiation, apoptosis, and homeostasis (48–50). In this study, we identified a relatively complete canonical TGF-β1 signaling pathway from the genome sequence of Nile tilapia. Notably, functional domains or motifs, and tertiary structures of TGF-β1 signaling components including TGF-β1, TGFBR1, TGFBR2, Smad2, Smad3, and Smad4 are well conserved in Nile tilapia and mammals. The evolutionary conservation of TGF-β1 signaling components was confirmed by phylogenetic analysis. These results provide evidence that Nile tilapia as one of the key species in teleost possesses an evolutionarily conserved TGF-β1 signaling pathway.

In mammals, TGF-β1 inhibits proliferation mainly by interacting with the receptors TGFBR2/TGFBR1 and activating the downstream canonical Smad signaling, including the recruitment, activation, and phosphorylation of Smad2/3, Smad complex formation, nuclear transport and Smad-DNA binding (9, 51). Activating downstream Smad molecules can also inhibit the expression of c-myc and promote the expression of cell cycle inhibitory proteins p21 and p15, resulting in the arrest of the cell cycle, indicating that TGF-β1 has the effect of inhibiting cell proliferation (8, 10, 52). Especially in the early stage of tumor formation, TGF-β1 can inhibit the process of tumor by inhibiting cell proliferation, blocking the cell cycle, inducing apoptosis and the expression of related factors (10, 52, 53). MBL, as a key and pleiotropic functional molecule, can interact with various types of cells and play an important regulatory function in cell proliferation, such as T cells and monocytes (11, 12). However, whether MBL has a regulatory function on cell proliferation in teleost fish and its regulatory function is related to the TGF signaling pathway remains unclear. In Nile tilapia, we found that OnMBL was able to directly bind to head kidney MФ and reduce the proliferation of activated MФ. More importantly, OnMBL could significantly up-regulate the expression of TGF-β1 and activate the downstream signaling pathway of TGF-β1 to reduce cell proliferation. To the best of our knowledge, these results represent the first description of a regulatory mechanism for MBL in MФ proliferation in teleost fish. However, mammalian MBL has multiple receptors, such as calreticulin (CRT), gC1qR/p32, C1qRp, CD91 and CR1 (54–57). Nowadays, we still have not confirmed the direct and exact receptor of OnMBL in Nile tilapia that regulates MФ proliferation. Previously, we have demonstrated that OnMBL promotes opsonophagocytosis of MФ by interacting with CRT (29). Thus, whether OnMBL regulates the proliferation of MФ through the TGF-β1 pathway is closely related to CRT or other receptors, it still needs further study.

**Figure 8:**
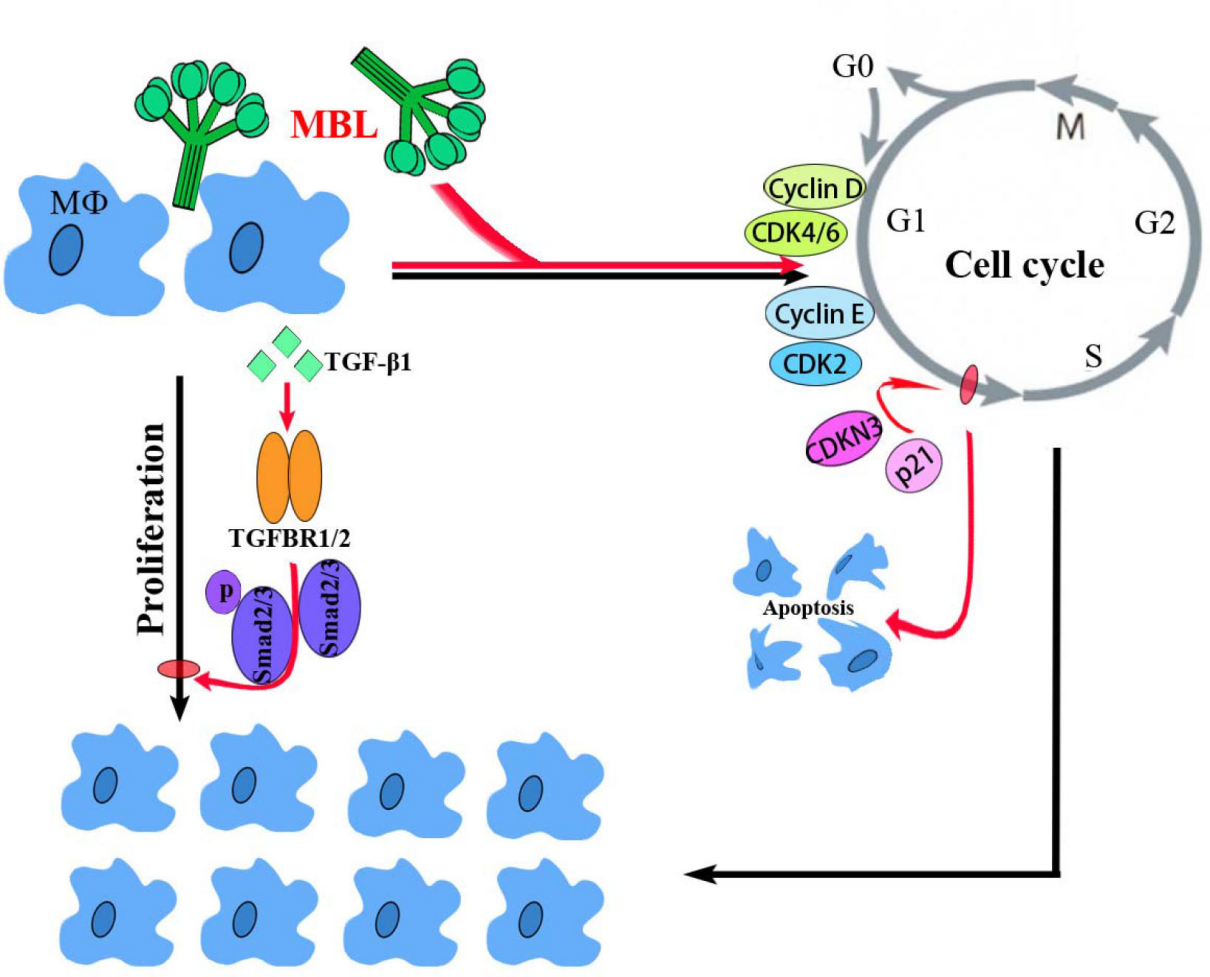
A proposed model of the functional characterization of OnMBL as a potent regulator in cell proliferation of teleost MФ. OnMBL could inhibit the proliferation of MФ by regulating the cell cycle, inducing apoptosis, and causing a large number of cells to stagnate in the G0/G1 phase. The inhibitory effect of proliferation in MФ might be related to the TGF-β1 signaling pathway activated by OnMBL.

In conclusion, the present study demonstrated a molecular mechanism of MBL in an early vertebrate Nile tilapia to regulate macrophage proliferation by the canonical TGF-β1 signaling pathway, as schematically illustrated in **Figure 8**. Through a series of analyses, OnMBL was found to bind directly to MФ and reduce the proliferation of activated-MФ by regulating the cell cycle, arresting a large number of cells in the G0/G1 phase, and increasing the probability of apoptosis. Meanwhile, the canonical and evolutionarily conserved TGF-β1 signaling pathway closely related to the regulation of cell proliferation was identified and analyzed. Interestingly, OnMBL could significantly up-regulate the expression of TGF-β1 and activate the downstream Smad-dependent signaling pathway of TGF-β1 to reduce cell proliferation, thereby maintaining the body’s cellular homeostasis. These results represent the first description regarding the regulatory mechanisms of MBL on cell proliferation in teleost fish, which provide a perspective on the understanding of the multiple function and evolutionary origins of C-type lectins in the host immune system.

## Data availability statement

The original contributions presented in the study are included in the article/**Supplementary Material**. Further inquiries can be directed to the corresponding authors.

## Ethics statement

The animal study was reviewed and approved by The University Animal Care and Use Committee of the South China Normal University (SCNU-SLS-2021-005).

## Author contributions

LM and XY designed experiments. LM and XY performed most of the experiments. HB and LQ contributed to laser confocal and flow cytometry analysis. JL, LQ, and QZ contributed to western blot, real-time PCR and bioinformatics analysis. LM wrote the manuscript. XY, SF and JY critically revised the manuscript. LM, XY, and JY obtained funding. XY, SF and JY administrative, technical, or other material support. All authors contributed to the article and approved the submitted version.

## Supplementary material

Supplementary Figure 1
**(A)** Purification of OnMBL. **(B)** Effects of OnMBL (50 μg/mL) on proliferation of *O. niloticus* MФ stimulated with or without 50 ng/mL hM-CSF were assessed by the CCK-8 assay. **(C)** The nucleotide and amino acid sequences of OnTGF-β1. **(D)** Domain prediction of Nile tilapia TGF-β1 pathway components, including TGFBR1, TGFBR2, Smad2, Smad3, and Smad4. **(E)** Analysis of grayscale values showing protein or phosphorylation levels of the indicated TGF-β1 pathway components in MФ with or without stimulation.

Supplementary Table 1 Primers used in this study
