# Supplementary material for: Mannose-binding lectin suppresses macrophage proliferation through TGF-β1 signaling pathway in Nile tilapia
Source: Front Immunol. 2023 May 16;14:1159577. doi: 10.3389/fimmu.2023.1159577 (PMC10227430; doi:10.3389/fimmu.2023.1159577)
Supplement: Supplementary Figure 1 — (A) Purification of OnMBL. (B) Effects of OnMBL (50 μg/mL) on proliferation of O. niloticus MФ stimulated with or without 50 ng/mL hM-CSF were assessed by the CCK-8 assay. (C) The nucleotide and amino acid sequences of OnTGF-β1. (D) Domain prediction of Nile tilapia TGF-β1 pathway components, including TGFBR1, TGFBR2, Smad2, Smad3, and Smad4. (E) Analysis of grayscale values showing protein or phosphorylation levels of the indicated TGF-β1 pathway components in MФ with or without stimulation. [file DataSheet_1.docx]

**Supplementary material**


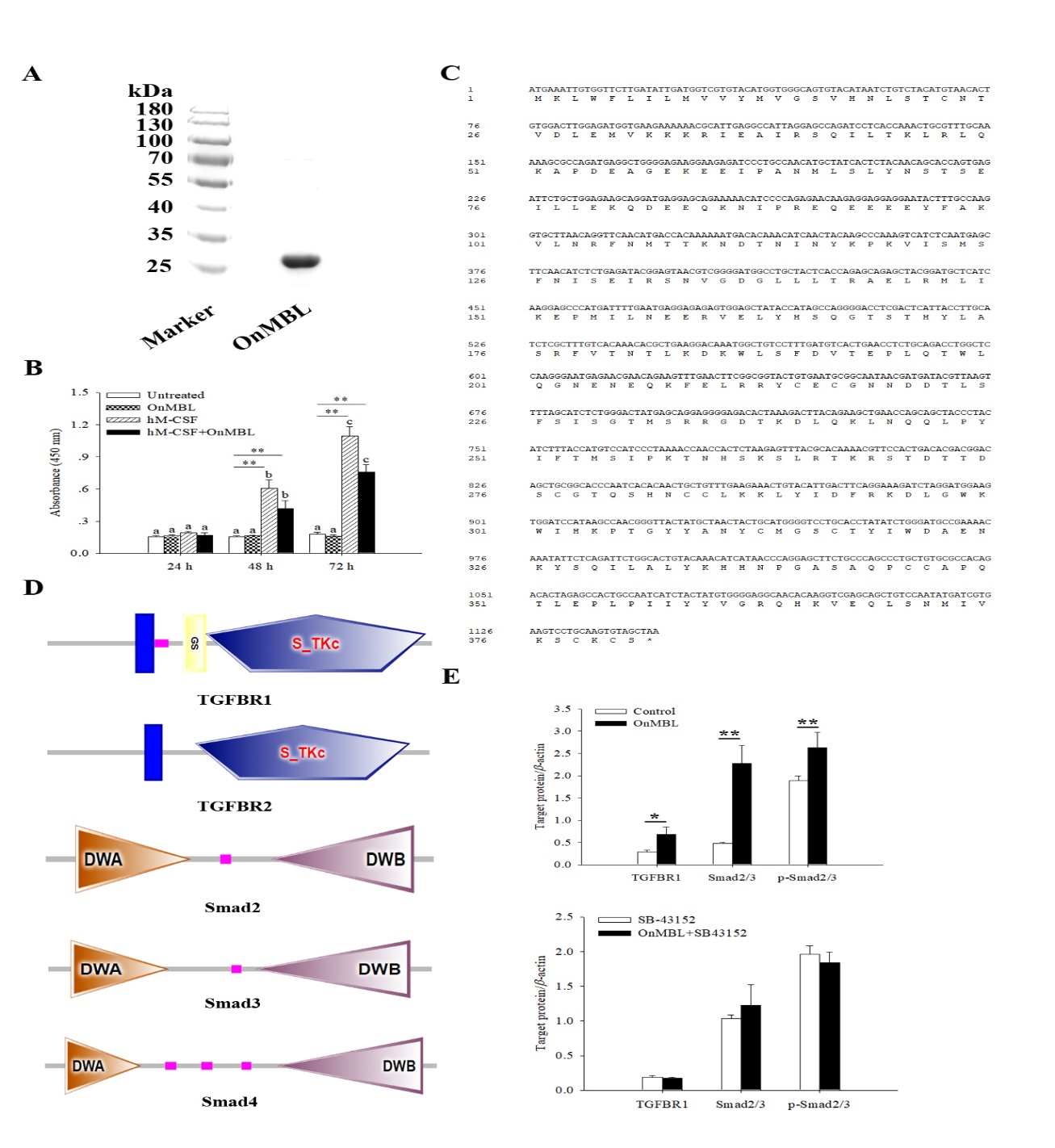


**Figure S1.** **(A)** Purification of OnMBL. **(B)** Effects of OnMBL (50 μg/mL) on proliferation of *O. niloticus* MФ stimulated with or without 50 ng/mL hM-CSF were assessed by the CCK-8 assay. **(C)** The nucleotide and amino acid sequences of OnTGF-β1. **(D)** Domain prediction of Nile tilapia TGF-β1 pathway components, including TGFBR1, TGFBR2, Smad2, Smad3, and Smad4. **(E)** Analysis of grayscale values showing protein or phosphorylation levels of the indicated TGF-β1 pathway components in MФ with or without stimulation.

**Table 1.** Primers used in this study

| Primers | Nucleotide Sequence (5’-3’) | Purpose |
| --- | --- | --- |
| qKi-67-F | CTGACTCTCCATTACGCAAGG | qRT-PCR |
| qKi-67-R | TTTAGCAAGCCGATGACG | qRT-PCR |
| qMaf-F | CCACCACACAGGGGCACACC | qRT-PCR |
| qMaf-F | GCTCGTCCGTGAACCGCTCT | qRT-PCR |
| qCyclin D1-F | GAGGATAGTCGCTGCTTGGATGT | qRT-PCR |
| qCyclin D1-R | CAGATGGTTCTTCTTCGTGGGTT | qRT-PCR |
| qCyclin D2-F | CGAGCACATCGTGAGAAGGC | qRT-PCR |
| qCyclin D2-R | GCACAGAGGGCAATGAAGGTC | qRT-PCR |
| qCyclin E2-F | GCCCAGGACTACTTTGACCGC | qRT-PCR |
| qCyclin E2-R | CTGGATGTCCCACAGGTCGC | qRT-PCR |
| qCDK2-F | CCGTTGACATCTGGAGTCTTGGA | qRT-PCR |
| qCDK2-R | CGGTCTCGTCAGGCGTTCC | qRT-PCR |
| qCDK4-F | TACCGACCTCCTGAAGTCCTGC | qRT-PCR |
| qCDK4-R | CAAAGGTTTCCGTCTGAACATCTC | qRT-PCR |
| qCDK6-F | GCTCCAGTCAAGTTACGCTACACC | qRT-PCR |
| qCDK6-R | GCCAGTCTTCTGCCGAGGGT | qRT-PCR |
| qp21-F | ACTGGCGAGGTCTGGAGC | qRT-PCR |
| qp21-R | CAGCCTGTCTGCGTTTCC | qRT-PCR |
| qp27-F | CAGCCTCACAGCAGACCGA | qRT-PCR |
| qp27-R | GCCACCATCGCTACTACGC | qRT-PCR |
| qCDKN3-F | GAACGACAGGAGGACGGTGAT | qRT-PCR |
| qCDKN3-R | GCTTTGTTTGGCGTCATTGTG | qRT-PCR |
| qBax-F | GAGCAAGGTGGCTGGGAGG | qRT-PCR |
| qBax-R | TGCGAATGACAAGAACAGTGGTAAG | qRT-PCR |
| qBcl-2-F | ACGCAGGCATCCACAGAGTC | qRT-PCR |
| qBcl-2-R | TCTATCACCTCGGCGAACCTC | qRT-PCR |
| qFasL-F | TCTCAGCAGAAGATACAGGTCG | qRT-PCR |
| qFasL-R | TGCCTCTAAGAACAGAATGCGTC | qRT-PCR |
| qFAIM-F | CGCACTTCATTGTCGGC | qRT-PCR |
| qFAIM-R | TCGCCATCCAGCAGTAAA | qRT-PCR |
| qCaspase-3-F | ATCACAGCAACTCAGCCTCTT | qRT-PCR |
| qCaspase-3-R | GGTTTTCCCACCAGTGATTTA | qRT-PCR |
| TGF-β1-F | AACACTGTGGACTTGGAGAT | Full cDNA |
| TGF-β1-R | GACCTTGTGTTGCCTCC | Full cDNA |
| ETGF-β1-F | CCGGAATTCAACACTGTGGACTTGGAGAT | Protein expression |
| ETGF-β1-R | CCGAAGCTTGACCTTGTGTTGCCTCC | Protein expression |
| qTGF-β1-F | CTGTGAATGCGGCAATAACG | qRT-PCR |
| qTGF-β1-R | TGTCTCCCCTCCTGCTCATAGTC | qRT-PCR |
| qTGFBR1-F | AACTCGCAGACGGCTCACG | qRT-PCR |
| qTGFBR1-R | CGCCCAGACTGTAGACTGTCAAACC | qRT-PCR |
| qTGFBR2-F | TCCCTGTGGTAGTGGTTAGTTTGG | qRT-PCR |
| qTGFBR2-R | ACCTTCTCCCTGAGGACCCA | qRT-PCR |
| qSmad2-F | AGACCTTCACAGCCACCACG | qRT-PCR |
| qSmad2-R | CCTCTGGTAGTGGTAAGGGTTGAT | qRT-PCR |
| qSmad4-F | CACCGCCTCACTCAAATCCC | qRT-PCR |
| qSmad4-R | CCACTGTGCTGCGGTCCTG | qRT-PCR |
| *β*-actin-F | CGAGAGGGAAATCGTGCGTGACA | Control, qRT-PCR |
| *β*-actin-R | AGGAAGGAAGGCTGGAAGAGGGC | Control, qRT-PCR |
